# Early versus late initiation of renal replacement therapy in patients with acute kidney injury-a systematic review & meta-analysis of randomized controlled trials

**DOI:** 10.1186/s12882-017-0486-9

**Published:** 2017-02-28

**Authors:** Girish Chandra Bhatt, Rashmi Ranjan Das

**Affiliations:** 1 Department of Pediatrics, All India Institute of Medical Sciences (AIIMS), Room no.18, OPD Block, Bhopal, Madhya Pradesh 462024 India; 2 Department of Pediatrics, All India Institute of Medical Sciences (AIIMS), Bhubaneswar, Odisha India

**Keywords:** Acute kidney Injury (AKI), Renal replacement therapy (RRT), Timing

## Abstract

**Background:**

Acute kidney injury (AKI) is a common complication in the critically ill patients and associated with a substantial morbidity and mortality. Severe AKI may be associated with up to 60% hospital mortality. Over the years, renal replacement therapy (RRT) has emerged as the mainstay of the treatment for AKI. However, the exact timing of initiation of RRT for better patient outcome is still debatable with conflicting data from randomized controlled trials. Thus, a systematic review and meta-analysis was performed to assess the impact of “early” versus “late” initiation of RRT.

**Methods:**

All the published literature through the major databases including Medline/Pubmed, Embase, and Google Scholar were searched from 1970 to October 2016. Reference lists from the articles were reviewed to identify additional pertinent articles. Retrieved papers concerning the effect of “early/prophylactic” RRT versus “late/as and when required” RRT were reviewed by the authors, and the data were extracted using a standardized data collection tool. Randomized trials (RCTs) comparing early initiation of RRT or prophylactic RRT with late or as and when required RRT were included. The primary outcome measures were all cause mortality and dialysis dependence on day 90. The secondary outcome measures were: length of ICU stay, length of hospital stay, recovery of renal function and adverse events.

**Results:**

Of the 547 citation retrieved, full text of 44 articles was assessed for eligibility. Of these a total of 10 RCTs with 1,636 participants were included. All the trials were open label; six trials have unclear or high risk of bias for allocation concealment while four trials have low risk of bias for allocation concealment. There was a variable definition of early versus late in different studies. Thus, the definition of early or late was taken according to individual study definition. Compared to late RRT, there was no significant benefit of early RRT on day 30 mortality [6 studies; 1301 participants; RR, 0.92;95% CI: 0.76, 1.12); day 60 mortality [3 trials;1075 participants; RR, 0.94; 95% CI: 0.78, 1.14)]; day 90 mortality [3 trials; 555 participants; RR,0.94;95% CI: 0.67, 1.33)]; overall ICU or hospital mortality; dialysis dependence on day 90 [3 trials; (RR, 1.06; 95% CI:0.53, 2.12)]. There was no significant difference between length of ICU or hospital stay or recovery of renal functions. A subgroup analysis based on modality of RRT or mixed medical and surgical vs. surgical or based on severity of illness showed no difference in outcome measure. The trials with high or unclear risk of bias for allocation concealment showed benefit of early RRT (RR, 0.74; 95% CI: 0.59, 0.91) while the trials with low risk of bias for allocation concealment showed no difference in the mortality (RR, 1.02; 95% CI: 0.89, 1.17). Grade evidence generated for most of the outcomes was “*low quality*”.

**Conclusion:**

This updated meta-analysis showed no added benefit of early initiation of RRT for patients with AKI. The grade evidence generated was of “low quality” and there was a high heterogeneity in the included trials.

**PROSPERO registration number:**

CRD42016043092.

**Electronic supplementary material:**

The online version of this article (doi:10.1186/s12882-017-0486-9) contains supplementary material, which is available to authorized users.

## Background

Acute kidney injury (AKI) is a common complication in the critically ill patients and associated with a substantial morbidity and mortality [1–3]. Severe AKI may be associated with up to 60% hospital mortality [4]. Over the years, renal replacement therapy (RRT) has emerged as the mainstay of the treatment for AKI. Intermittent hemodialysis (IHD), peritoneal dialysis (PD) and continues renal replacement therapy (CRRT) are various modalities to conduct RRT. Early initiation of RRT helps in the removal of uremic toxins, allow fluid and electrolyte balance and prevent life threatening complications such as metabolic encephalopathy, hyperkalemia, pulmonary oedema [5].

The timing of initiation of RRT for better patient outcome is still debatable with conflicting data from randomized controlled trials [6–9]. Two meta-analysis concluded that early RRT improves survival in critically ill patients [10, 11] . However, a recent meta-analysis [12] concluded that “early” initiation of RRT in critical illness complicated by AKI does not improve patient survival or confer reductions in intensive care unit (ICU) or hospital length of stay (LOS). This meta-analysis included both RCTs, and cohort studies. Moreover, after publication of this meta-analysis, two large studies have been published. We conducted an updated systematic review including RCTs and Quasi-RCTs (no observational studies) to support or refute the earlier evidence on the initiation of early versus late RRT. We have also performed a robust subgroup and sensitivity analysis as well as graded the quality of evidence and strength of recommendations by using GRADE approach which is lacking in previous systematic reviews and metanalysis.

### Objective

To evaluate the impact of “early” versus “late” initiation of RRT.

## Methods

The review has been registered at the PROSPERO register: CRD42016043092

### Type of studies

Randomized controlled trials and quasi-randomized trials (RCTs) were included.

### Participants

Hospitalized patients with AKI were included. Patients with preexisting chronic kidney disease (estimated glomerular filtration rate [GFR] <30 mL/min) on long term dialysis, previous renal replacement therapy, AKI resulting from vascular malformations (occlusion of the renal artery), glomerulonephritis, interstitial nephritis, vasculitis, post-renal obstruction, hemolytic uremic syndrome (HUS) or thrombotic thrombocytopenic purpura, post renal transplant AKI and confirmed or suspected pregnancy, malignancy and HIV were excluded.

### Interventions

The interventions consist of administration of early/prophylactic or as and when required/late RRT in patients with AKI. The definition of early and late RRT was taken as described in the individual study.

### Types of outcome measures

*Primary outcomes*

1. Mortality rate
2. Dialysis Dependence at 3 month

*Secondary outcomes*

1. Length of ICU stay
2. Length of hospital stay
3. Recovery of renal function
4. Adverse events

### Search methods for identification of studies

Cochrane Central Register of Controlled Trials (CENTRAL), PubMed/MEDLINE, Google Scholar, Cochrane renal group were searched from 1970 to October 2016. Following search strategy was applied: (((((((((renal replacement therapy) OR Renal Dialysis) OR dialysis) OR Hemodialysis) OR Hemodiafiltration) OR Hemofiltration)) AND (((((acute kidney injury) OR Acute Renal Injury) OR Acute Renal Insufficiency) OR Acute Renal Failure) OR Acute Kidney Failure)) AND (((((((timing) OR time) OR Initiation) OR start) OR early) OR Earlier) OR Late)) AND ((randomized controlled trial) OR Controlled Clinical Trial). To identify unpublished trial results, we searched the US National Institutes of Health, Department of Health and Human Services trials registry (http://www.clinicaltrials.gov/) and the WHO International Clinical Trials Registry Platform (ICTRP) trial registry.

### Data extraction

Data was extracted using a pilot tested data extraction form. Two authors independently extract data including author, type of participants, exposure and intervention (modality of RRT, timing), results (clinical outcomes and adverse events).

### Risk of bias (quality) assessment

Two review authors (GC and RD) independently assessed the methodological quality of the selected trials by using Cochrane risk of bias tool [13].

### Strategy for data synthesis

The data from various studies was pooled and expressed as mean difference (MD) with 95% confidence interval (CI) in case of continuous data, and risk ratio (RR) with 95% CI in case of categorical data. *P*-value <0.05 was considered significant. Heterogeneity was assessed by I-squared statistics. In case of high level heterogeneity (>50%), we tried to explore the cause. A fixed effects model was initially conducted. If, significant heterogeneity was found between trials, potential sources of heterogeneity were considered and where appropriate, a random effects model was used. RevMan (Review Manager) version 5.3 was used for all the analyses.

### Subgroup analysis

We performed the following subgroup analysis:

1. Surgical versus mixed medical admission.
2. Modality of RRT
3. Severity of illness
4. Risk of bias for allocation concealment

### Publication bias

This was looked by construction of the inverted funnel plot as suggest by Egger et al.[14].

### Grade of evidence

For assessment of the quality of evidence we used GRADE Profiler software (version 3.2). The software uses five parameters for rating the quality of evidence. The parameters used were - limitations to design of randomized controlled trials, inconsistency of results or unexplained heterogeneity, indirectness of evidence, imprecision of results, and publication bias. The rating was done as – no, serious, and very serious limitation.

## Results

### Description of the studies

**Fig. 1:**
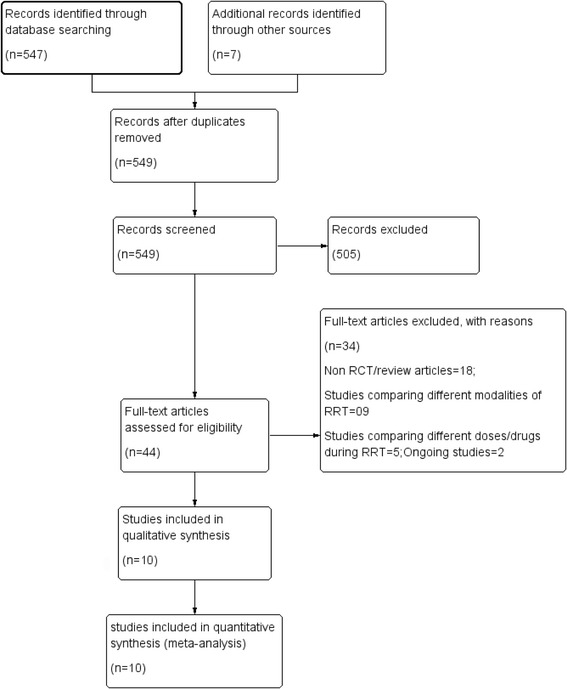
PRISMA flow diagram

Of the 547 citation retrieved, full text of 44 articles was assessed for eligibility (Fig. 1). Of these a total of 10 RCTs with 1,672 participants were included. Thirty three studies were excluded due to following reasons: Non RCT/review articles (*n* = 18), comparing different modalities of RRT (*n* = 09), comparing different doses/drugs during RRT (*n* = 05); Ongoing studies (*n* = 02). All the trials were open label with most of the trials having unclear or high risk of bias for allocation concealment. There was a variable definition of early versus late in different studies. Thus, the definition of early or late was taken according to individual study definition. A summary of the studies is provided in Table 1.

**Table 1:** Characteristics of the included studies

Study author	Setting, Country	Participants	Intervention	Outcomes measured	Comments
Pursnani 1997 [18]	India	Number: 35 (Early RRT = 18; Late RRT = 17 Age: Adults Inclusion criterion: cases of acute tubular necrosis with creatinine <7 mg% and blood urea <120 mg% Exclusion criterion: cases with creatinine >7 mg% and urea blood urea >120 mg%.	Early RRT = Prophylactic hemodialysis was performed Late RRT = Conservatively managed	Mortality, complications and hospital stay	Method of randomization not given. Blinding and allocation concealment not clear. Small sample size
Bouman 2002 [7]	Two Centres (Intensive care unit), Netherlands;	Number: 106 patients (Early RRT = 70; late RRT = 36 Age: 18-90 years Inclusion: Critically ill patients with circulatory and respiratory failure, developing early oliguric ARF, Exclusion: Pre-existing renal disease	Early RRT = Hemofiltration started within 12 h of inclusion Late RRT = Hemofiltration started when patient fulfilled conventional criterion for renal replacement therapy such as plasma urea level of 40 mmol/L, potassium of 6.5 mmol/L or severe pulmonary edema.,	Survival at day 28 after inclusion and recovery of renal function, ICU survival, hospital survival, duration of mechanical ventilation, length of ICU stay, and length of hospitalization.	Open label. Allocation concealment not clear. Three treatment strategies were compared viz early high volume hemofiltration, early low volume hemofiltration and late low volume hemofiltration. Small sample size.
Durmaz 2003 [16]	Single centre (In-patients undergoing coronary artery bypass surgery) Turkey	Number: 44 (Early RRT = 21;Late RRT = 23 Age: Adult patients Inclusion: Post cardiac surgery Exclusion: Age less than 18 years; chronic dialysis	Early RRT = Pre-operative prophylactic hemodialysis was performed if serum creatinine ≥2.5 mg/dl Late RRT = Hemodialysis was performed only if postoperative acute renal failure was seen (defined as urine output of less than 400 mL in a 24-h period, a 50% increase in serum creatinine from base line, or need for dialysis).	Overall 30 day mortality; mean decrease in serum creatinine, potassium and BUN levels; average length of stay in cardiac ICU, average length of postoperative in-hospital stay.	Quasi randomized trial. Small sample size.
Sugahara 2004 [19]	Single Centre (in-patient) Japan	Number: 28 (Early RRT = 14; Late RRT = 14) Age Adults: Inclusion: Post cardiac surgery Exclusion: Pregnancy, Bilirubin >5 mg/dl, mental disorders, Cancer, Early recovery of urine output i.e >30 ml/kg prior to RRT	Early RRT = Dialys started if ourly urine output <30 ml/kg for 3 h or daily urine output ≤750 ml . Late RRT = Dialysis started if hourly urine output <20 ml/kg for 2 consecutive hours or daily urine output ≤500 ml.	Overall 14 days mortality, changes in the blood pressure, changes in serum creatinine, changes in urine output.	Open label randomized controlled trial. Allocation concealment not clear. Small sample size.
Payen 2009 [5]	Multicentric (Intensive care units), France	Number: 76 (Early RRT = 37;Control group = 39 Age: Adult patients Inclusion: Multisystem/severe sepsis. Exclusion:	Early RRT = Hemofiltration started if, clinically identified focus on infection associated with at least 2 systemic inflammatory response syndrome criteria and one or more sepsis-induced organ failures within the 24 h before inclusion, plus a Simplified Acute Physiology II score between 35 and 63. Control group = standard therapy	Overall 28 day mortality; occurrence or worsening of sepsis induced organ failure (SOFA score), length of ICU stay, duration of mechanical ventilation, ionotropic support,,measurement s of cytokines, adverse events.	Open label. Allocation concealment unclear. Authors concluded that in septic patients, hemofiltration with an ultrafiltration rate of 2 L/h did not limit organ failure
Jamale 2013 [6]	Single centre (Intensive care unit), India	Number: 208 (Early RRT = 102;late RRT = 106) Age: Adult patients Inclusion: Patients with severe community acquired AKI with increasing serum urea nitrogen and creatinine levels. Exclusion: Requirement of urgent dialysis for life-threatening uremic complications (ie, treatment-refractory hyperkalemia and fluid overload, alteration of higher mental function attributable to uremia, and pericarditis), patients who received dialysis therapy before evaluation, and who were judged to be in the recovery phase.	Early RRT = initiation of dialysis therapy if serum urea nitrogen level increased to >70 mg/dL and/or creatinine level increased to >7 mg/dL irrespective of complications till recovery of renal functions. Late RRT = Dialysis therapy was initiated only if participants developed complications such as treatment-refractory hyperkalemia, volume overload, and acidosis, uremic nausea and anorexia leading to inability to maintain nutrient intake until recovery	Overall 3 months mortality, Dialysis dependence at 3 months, increase in urine output, decrease in blood urea nitrogen and creatinine, days to renal recovery, adverse events such as bleeding, number of catheter related complications including infections, number of episodes of intradialytic hypotension, requirement of blood products for transfusion,	Open label trial. Event rate (mortality) was less than predicted. Study population included community acquired AKI (different from usual AKI population). Use of intermittent dialysis (not continuous) as a modality of RRT.
Combes 2015 [15]	Multicentric (Postoperative ICU) France	Number = 224 (Early RRT = 112; Late RRT/standard care = 112) Age: Adult patients Inclusion criterion: Post cardiac surgery with persistent postoperative shock requiring high dose catecholamines within 24 h following surgery. Exclusion criterion: Younger than 18 years old; pregnant; previously enrolled in this or other trials evaluating mortality; on chronic hemodialysis prior to heart surgery; weight greater than 120 kg; moribund state (defined as Simplified Acute Physiology Score (SAPS) II greater than 90); or those for whom active therapeutics were withheld or withdrawn.	Early RRT = High volume hemofiltration for 48 h followed by standard-volume continuous venovenous hemodiafiltration (CVVHD) till recovery of renal function. Late RRT/control group = Supportive management was provided and CVVHDF (CVVHDF (if serum creatinine >4 mg/dl] or threefold increase of preoperative values, or urine output < 0.3 ml/kg/h for 24 h despite adequate fluid resuscitation; serum urea > 36 mmol/L, or life-threatening hyperkalemia).	Overall mortality on day 30, day 60 and day 90, ICU and hospital length of stay; Day 30 duration of catecholamine infusion, RRT and mechanical ventilation; numbers of catecholamine, RRT and mechanical ventilation free days; Sequential Organ Failure Assessment (SOFA) score until Day 30, percentage of patients with renal recovery and adverse events.	Open label trial. Allocation concealment not done. Trail was prematurely terminated after only 2/3^rd^ of the calculated enrollments. Only post cardiac surgery patients were enrolled.
Wald 2015 [17]	Multicentric (Intensive care unit) Canada	Number = 101 (Early RRT group = 48; Late RRT/control group = 52) Age : Adult patients Inclusion criterion: presence of severe AKI (defined by the presence of two of the following three criteria: (i) a twofold increase in serum creatinine from baseline, (ii) urine output 0.6 ml/kg in the preceding 12 h, or (iii) whole-blood NGAL ≥ 400 ng/ml); [2] the absence of urgent indications for RRT initiation (defined as serum potassium ≤ 5.5 mmol/l and serum bicarbonate ≥ 15 mmol/l); and [3] low likelihood of volume-responsive AKI (defined as central venous pressure ≥ 8 mm Hg). Exclusion criterion: Lack of commitment to ongoing life support, including RRT; presence of an intoxication requiring extracorporeal removal; RRT within the previous 2 months; clinical suspicion of renal obstruction, rapidly progressive glomerulonephritis, or interstitial nephritis; pre-hospitalization estimated glomerular filtration rate o 30 ml/min per 1.73 m2; and the passage of 4 48 h since doubling of baseline serum creatinine.	Early RRT = patients that fulfilled inclusion criterion were started RRT modality based on current best practice guidelines till death, recovery of renal functions or changes in goals of care. Late RRT/Control group = Supportive management was provided and RRT initiated once following condition developed: Serum potassium > 6.0 mmol/l, serum bicarbonate < 10 mmol/l, or PaO2/ FiO2 < 200 with infiltrates on chest radiograph compatible with pulmonary edema. RRT was to continue until patient death, change in goals of care, or recovery of kidney function. Modality selection was based on physicians discretion (IDH, SLED or CRRT).	Proportion of patients in each arm who commenced RRT within the protocol-specified window (≤12 and 4 12 h), the proportion of patients successfully consented among those fully eligible (feasibility target ≥ 50%),the proportion of patients followed to day 90 (feasibility target ≥ 95%), and serious adverse events.	Open label, parallel feasibility randomized controlled trial. Allocation concealment not clear. Small sample size
Gaudry 2016 [8]	Multi-centric (Intensive care units) France	Number: 620 (Early RRT = 312; Late RRT/control group = 308) Age: Adult patients Inclusion criterion: Patient with severe acute kidney injury (KDIGO stage 3) requiring mechanical ventilation, catecholamine infusion or both and did not have potentially life threatening complications directly related to renal failure. Exclusion criterion: Age <18 years, a blood urea nitrogen level >112 mg per deciliter (40 mmol per liter), a serum potassium concentration > 6 mmol per liter (or greater than 5.5 mmol per liter despite medical treatment), a pH <7.15 in the context of either pure metabolic acidosis (partial pressure of arterial carbon dioxide [Pa co2] below 35 mm Hg) or mixed acidosis (Pa co2 of 50 mm Hg or more without the possibility of increasing alveolar ventilation), and acute pulmonary edema due to fluid overload responsible for severe hypoxemia requiring an oxygen flow rate greater than 5 l per minute to maintain a peripheral capillary oxygen saturation (Sp o2) greater than 95% or requiring a fraction of inspired oxygen (F io2) greater than 50% in patients receiving mechanical ventilation and despite diuretic therapy.	Early RRT = RRT started as soon as possible after randomization in order for it to be started within 6 h of documentation of stage 3 AKI. Late RRT = Renal-replacement therapy was initiated if one of the laboratory abnormalities defined in the exclusion criterion developed or if oliguria or anuria lasted for more than 72 h after randomization. The choice of the method of renal-replacement therapy (intermittent or continuous technique, duration and interval between sessions, device setting, and anticoagulation method) was left to the discretion of each study site and was prescribed and monitored according to national guidelines.	Overall mortality on day 60, receipt of renal-replacement therapy at least once with the delayed strategy; numbers of renal-replacement therapy–free days, dialysis catheter–free days, mechanical ventilation–free days, and vasopressor therapy–free days on day 28, Sepsis-related Organ Failure Assessment (SOFA) score at day 3 and day 7; the vital status at day 28; the length of stay in the intensive care unit and in the hospital; the proportion of patients with treatment limitations; the occurrence of nosocomial infections; and complications potentially related to acute kidney injury or renal replacement therapy.	Open label trial. Inadequate sample size..The patients in the trial have advance acute kidney injury i.e KDIGO stage 3 reducing generalizability of the study among different staging.
Zarbock 2016 [9]	Single centre (Intensive care unit) Germany	Number = 2319 (Early RRT = 112; Late RRT/control group = 119) Age: 18-90 years. Inclusion criterion: critically ill patients with AKI Kidney Disease: Improving Global Outcomes (KDIGO) stage 2 (2 times baseline or urinary output <0.5 mL/kg/h for 12 h) and plasma neutrophil gelatinase–associated lipocalin level higher than 150 ng/m. Exclusion criterion:	Early RRT: Continuous venovenous hemodiafiltration was initiated within 8 h of diagnosis of stage 2 AKI using KDIGO classification. Late RRT: Continuous venovenous hemodiafiltration was initiated within 12 h of diagnosing stage 3 AKI by KDIGO criterion or if any of the following absolute indications for RRT were present: serum urea level higher than 100 mg/dL; serum potassium level higher than 6 mEq/L and/or with electrocardiography abnormalities; serum magnesium level higher than 8 mEq/L (to convert to mmol/L, multiply by 0.5); urine production lower than 200 mL per 12 h or anuria (according to the KDIGO recommendations); and organ edema in the presence of AKI resistant to diuretic treatment.	Overall mortality on day 90, mortality on day 28 and day 60,clinical evidence of organ dysfunction determined by SOFA scores, recovery of renal functions, requirement of hemodialysis after day 28 and 60, duration of renal support, ICU and hospital length of stay and markers of inflammation.	Open label trial. Allocation concealment not clear. Limited generalizability as almost all patients recruited were surgical patients. Authors proposed that adequately powered multicenter trial is needed to confirm our results and establish the best time point for the initiation of RRT in critically ill patients with AKI.

### Primary outcome measure

**Fig. 2:**
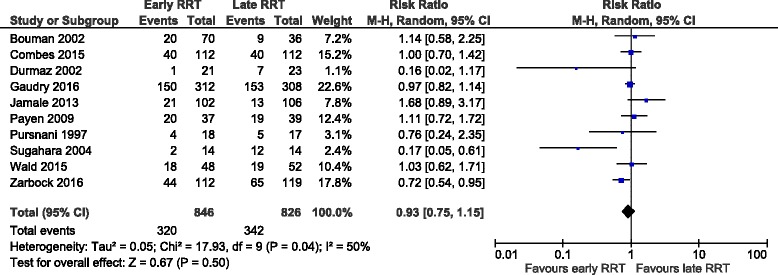
Forest plot showing overall mortality

Overall Mortality: Ten studies with 1672 participants reported 662 deaths. Compared with the patients assigned to late RRT, patients assigned to early RRT had 7% reduction in mortality rate. However, pooled results showed no significant difference between the two groups (RR, 0.93;95% CI: 0.75, 1.15) (Fig. 2). Since there was a significant heterogeneity (I^2^ = 50%;*p* = 0.17), we tried to explore the heterogeneity based on pre-specified subgroups analysis such as: Surgical versus mixed patients, severity of illness, modality of RRT and risk of bias for allocation concealment. We also performed a period wise mortality analysis to address the heterogeneity in the included trials.

Day 30 mortality: This was reported in 6 trials [5, 7–9, 15, 16] with 1301 participants. The pooled results showed 8% decrease in mortality with early initiation of RRT. However, there was no significant difference between the early and late RRT (RR, 0.92;95% CI: 0.68, 1.06], (Additional file 1: Figure S1 a)

Day 60 mortality: This was reported in 3 trials [8, 9, 15] with 1075 participants. The pooled results showed no significant difference between the two strategies (RR, 0.94; 95% CI:0.78, 1.14) (Additional file 1: Figure S1a).

Day 90 mortality: This was reported in three trials [9, 15, 17] with 555 participants. The pooled results showed no significant difference between the two strategies (RR, 0.94; 95% CI:0.67, 1.33) (Additional file 1: Figure S1a) .

Overall ICU mortality: Overall ICU mortality was reported in 3 trials [7, 15, 17]. Pooled mortality showed no significant reduction in ICU mortality with initiation of early RRT (RR, 1.08; 95% CI:0.84, 1.39) (Additional file 1: Figure S1a).

Overall hospital mortality: This was reported in 6 trials and the pooled results showed no significant difference between the mortality rates between the two groups (RR, 1.07; 95% CI: 0.81, 1.42) (Additional file 1: Figure S1a).

Dialysis Dependence at Day 90: 3 trials [6, 9, 17] reported dialysis dependence at 90 days in the two groups. Pooled data showed no significant difference between the two groups (RR, 1.06 95% CI: 0.53, 2.12) (Additional file 1: Figure S1c)

### Subgroup based on Surgical versus mixed patients

Overall 30 day mortality: 2 trials reported this outcome [15, 16]. Overall there was no significant difference in overall 30 day mortality (RR,0.51;95% CI: 0.09, 3.08.) (Additional file 1: Figure S1b)

Overall 60 day mortality: Only 1 trial [15] reported this outcome. Overall there was no significant difference between the two groups (RR, 1.14;95% CI: 0.83, 1.58)

Overall 90 day mortality: 1 trial [15] reported this outcome without any significant difference between the two strategies.

Overall ICU: There was no significant difference between ICU (RR,1.11;95% CI:0.82, 1.52) or hospital mortality (RR,1.01;95% CI:0.74, 1.36) in the surgical patients undergoing early versus late initiation of RRT.

### Subgroup analysis based on severity of illness

There was no significant difference in overall day 30 mortality (5 trials, 1257 patients;RR,0.91;95% CI:0.73, 1.15);day 60 mortality (3 trials, 1075 participants; RR, 0.90; 95% CI:0.64, 1.27); day 90 mortality (3 trial, 555 participants, RR, 0.90; 95% CI:0.49, 1.64), hospital mortality (RR, 1.12; 95% CI: 0.76, 1.65) and ICU mortality (RR, 1.12;95% CI:0.75, 1.68) in critically ill undergoing early RRT as compared to late RRT.

### Subgroup analysis based on modality of RRT

**Fig. 3:**
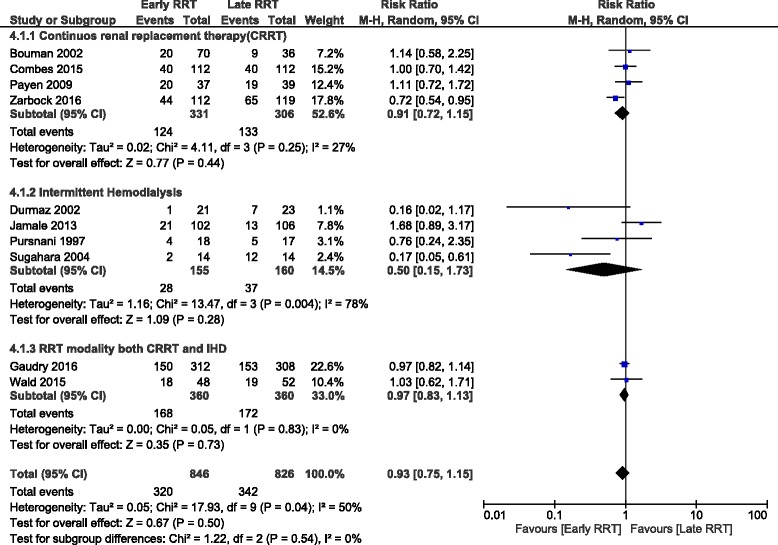
Forest plot showing subgroup analysis based on modality of RRT

There was no significant difference in overall day 30 mortality in the patients undergoing continuous renal replacement therapy (CRRT) (3 trials, 413 participants; RR, 0.90;95% CI:0.65, 1.26); patients undergoing intermittent hemodialysis (IHD) (1 trial,44 participants; RR, 0.16;95% CI:0.02, 1.17); patients undergoing either CRRT or IHD (1 trial,620 participants; RR, 0.95;95% CI:0.79, 1.14): day 60 mortality in patients undergoing CRRT (2 trials, 455 participants; RR, 0.93;95% CI:0.62, 1.38) or patients undergoing either CRRT or IHD (1 trial,620 participants; RR, 0.97;95% CI: 0.82, 1.14): day 90 mortality in patients undergoing CRRT (2 trials, 455 participants; RR, 0.92;95% CI:[0.56, 1.50) or patients undergoing either CRRT or IHD (1 trial,100 participants; RR, 1.03;95% CI:0.62, 1.71): Overall hospital mortality in the patients undergoing CRRT (2 trials, 330 participants; RR, 1.14;95% CI:0.88, 1.48) or IHD (1 trial,44 participants; RR, 0.16;95% CI:0.02, 1.17) and overall ICU mortality in the patients undergoing CRRT (2 trial, 330 participants; RR, 1.13;95% CI: 0.85, 1.49) or patients undergoing either CRRT or IHD (1 trial, 100 participants; RR, 0.88;95% CI: 0.47, 1.63) (Fig. 3)

### Subgroup analysis based on risk of bias for allocation concealment

**Fig. 4:**
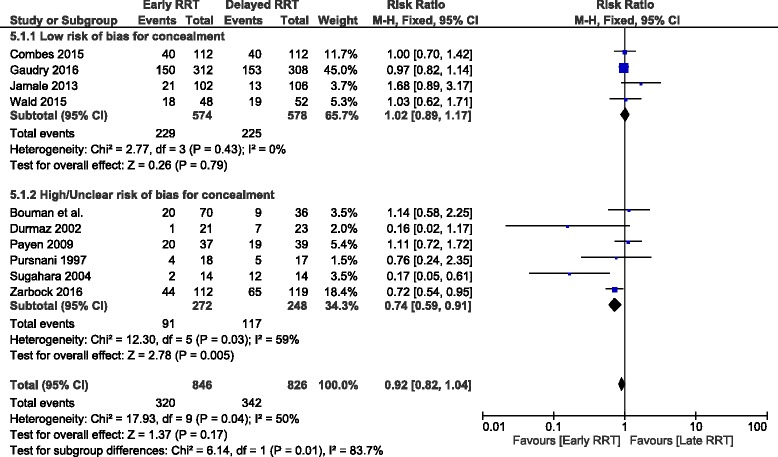
Forest plot showing subgroup based on risk of bias for allocation concealment

Six trials have low risk of bias for allocation concealment [6, 8, 9, 15, 17] while 4 [5, 7, 16, 18, 19] have unclear or high risk of bias for allocation concealment. There was a significant reduction in overall mortality in the patients assigned to early RRT in the studies with high or unclear risk of bias (RR, 0.74; 95% CI:0.59, 0.91) as compared to those with low risk of bias for allocation concealment (RR, 1.02;95% CI:0.89, 1.17) (Fig. 4)

### Secondary Outcomes

Length of ICU stay: Six studies reported this outcome [8, 9, 15–17]. Out of these, 5 trials reported this outcome as median (interquartile range) [7–9, 15, 17] and found no significant difference between ICU stay in the two groups. Another trial [16] reported a significant reduction in ICU stay in the patients undergoing early RRT as compared to late RRT (MD,-45.87;95% CI:-75.54,–16.20).

Length of Hospital stay: Seven trials reported this outcome [8, 9, 15–17] and 5 reported them as median (Interquartile range). In 4 trials there was no significant difference in the length of hospital stay between the two groups while 1 trial has shown significant difference between hospital stay in patients receiving early RRT. Two trials [7, 18] have given this outcome as mean (SD) and were pooled. The pooled results no difference in the length of hospital stay (MD,-3.62; 95% CI :-8.91, 16.16).

Recovery of renal function by day 90: 2 trials reported recovery of renal functions on day 90 [9, 15]. Pooled data showed no significant difference between two groups (RR, 1.04;95% CI:0.80, 1.35) (Additional file 1: Figure S1d).

### Adverse events

**Fig. 5:**
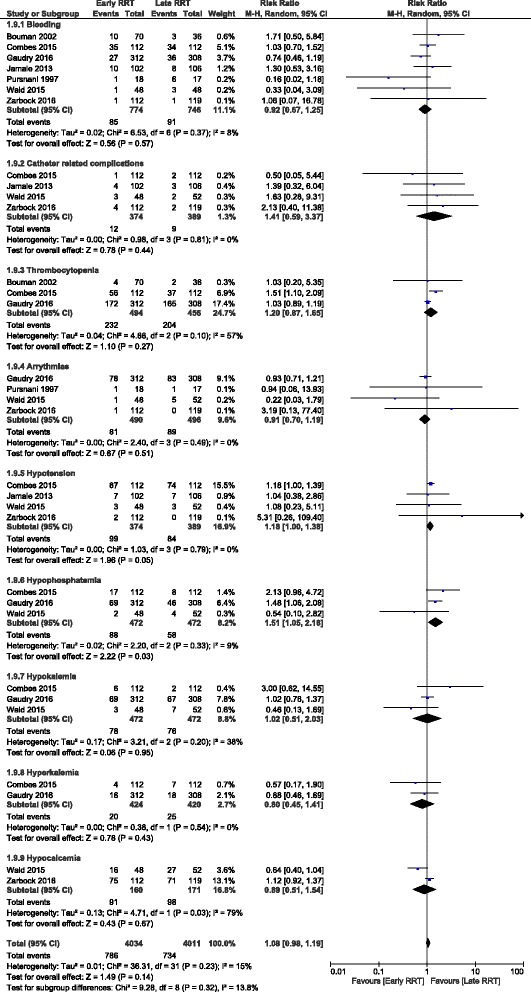
Forest plot showing adverse events

Bleeding: 7 trials with 1520 participants reported this outcome [6–9, 15, 17, 18]. On pooling the data no significant difference in the adverse event was observed between the two groups (RR, 0.92;95% CI:0.67, 1.25) (Fig. 5).

Catheter related complications: Four trials reported this outcome [6, 9, 15, 17]. The pooled results showed no significant difference between the two groups (RR, 1.41: 95% CI: 0.59, 3.37) with point estimate favouring late strategy (Fig. 5).

Thrombocytopenia: 3 trials reported this outcome [8, 9, 17, 18] and the pooled results showed no significant difference between the two strategies (RR,1.20:95% CI:0.87, 1.65) (Fig. 5).

Arrhythmias: 4 trials reported this outcome [8, 9, 17, 18] and the pooled results showed no significant difference between the two groups (RR, 0.91;95% CI: 0.70, 1.19) (Fig. 5).

Hypotension: 4 trials reported this outcome [6, 9, 15, 17] reported this outcome. Pooled results showed no significant difference with point estimate favouring late RRT (RR, 1.18; 95% CI: 1.00, 1.38) (Fig. 5).

Electrolyte abnormalities: There was no significant difference between the two strategies with respect to hypokalemia (RR, 1.02;95% CI: 0.51, 2.03), hyperkalemia (RR, 0.80;95% CI: 0.45, 1.41) and hypocalcaemia (RR, 0.89;95% CI:0.51, 1.54). Hypophosphatemia was seen more in patients undergoing early dialysis (RR, 1.51; 95% CI: 1.05, 2.18) (Fig. 5).

### Publication bias

**Fig. 6:**
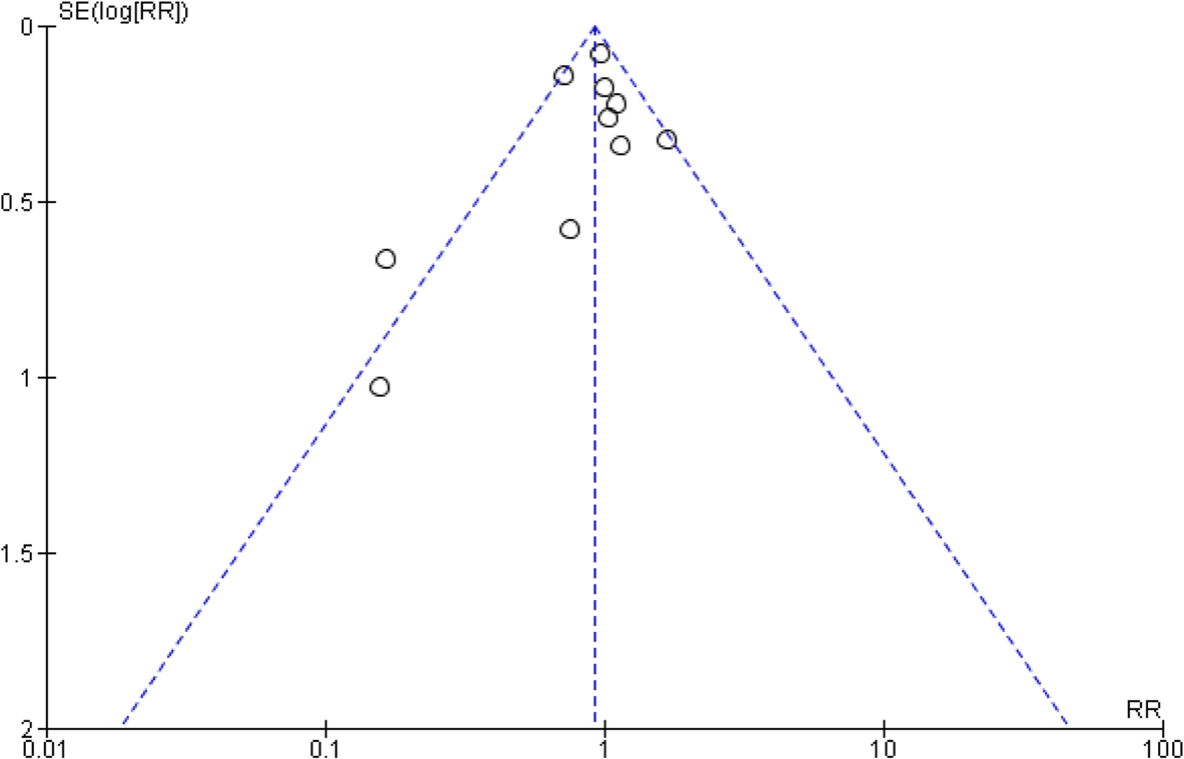
Funnel plot

To assess whether there was a bias in the published literature, funnel plot was constructed using the MD and 1/SE values obtained from trials measuring one of the primary outcome (overall mortality). In the absence of a publication bias, such a plot is expected to have a shape resembling an inverted funnel. From the funnel plot generated, the possibility of publication bias in the analysis is less (Fig. 6).

### Grade of evidence

The evidence generated was of “low quality” for all the primary outcomes (GRADE Table 2).

**Table 2:** Grade of evidence for primary outcomes

Early RRT compared to Late RRT for aki					
Outcomes	No of participants (studies)	Quality of evidence (GRADE)	Relative effect (95% CI)	Anticipated absolute effects	
	Follow up			Risk with Late RRT	Risk difference with Early RRT (95% CI)
Overall mortality	1672 (10 studies)	⊕⊝ ⊕ ⊝ Low^a,b^ due to risk of bias, inconsistency	RR 0.93 (0.75 to 1.15)	study population	
				414 per 1000	29 fewer per 1000 (from 104 fewer to 62 more)
Day 30 mortality	1301 (6 studies)	⊕⊝ ⊕ ⊝ Low^a,b^ due to risk of bias, inconsistency	RR 0.85 (0.6 to 1.2)	study population	
				430 per 1000	65 fewer per 1000 (from 172 fewer to 86 more)
Day 60 mortality	1075 (3 studies)	⊕⊝ ⊕ ⊝ Low^a,b^ due to risk of bias, inconsistency	RR (0.64 to 1.27)	study population	
				473 per 1000	47 fewer per 1000 (from 170 fewer to 128 more)
Day 90 mortality	555 (3 studies)	⊕⊝ ⊕ ⊝ Low^a,b^ due to risk of bias, inconsistency	RR (0.49 to 1.64)	study population	
				449 per 1000	45 fewer per 1000 (from 229 fewer to 287 more)
Overall ICU mortality	430 (3 studies)	⊕⊝ ⊕ ⊝ Low^a,b^ due to risk of bias, inconsistency	RR (0.75 to 1.68)	study population	
				350 per 1000	42 fewer per 1000 (from 87 fewer to 238 more)
Overall Hospital mortality	713 (6 studies)	⊕⊝ ⊕ ⊝ Low^a,b^ due to risk of bias, inconsistency	RR (0.7 to 1.68)	study population	
				298 per 1000	24 fewer per 1000 (from 89 fewer to 203 more)
Dialysis dependence at day 90	539 (3 studies)	⊕⊝ ⊕ ⊝ Low^a,b^ due to risk of bias, inconsistency	RR (0.51 to 2.22)	study population	
				54 per 1000	3 more per 1000 (from 27 fewer to 66 more)

The basis for the assumed risk (e.g. the median control group risk across studies) is provided in footnotes. The corresponding risk (and its 95% confidence interval) is based on the assumed risk in the comparison group and the relative effect of the intervention (and its 95% CI). CI: Confidence interval; RR: Risk ration; GRADE Working Group of evidence

High quality: Further research is very unlikely to change our confidence in the estimate of effect

Moderate quality: Further research if likely to have an important impact on our confidence in the estimate of effect and may change the estimete

Low quality: Further research if very likely to have an important impact on our confidence in the estimate of effect and may change the estimete

Very low quality: We ae very uncertain about the estimate

^a^Heterogeneity

^b^The risk was increased or decreased

## Discussion

### Summary of Evidence

After an extensive search of literature we could find 10 trials to be eligible for inclusion. Our results indicates that in patients with AKI there is no benefit of early initiation of renal replacement therapy on overall mortality, dialysis dependence on day 90, length of ICU or hospital stay and renal recovery on day 90. There was no significant difference in the adverse events between early and late group except for hypophosphatemia which was seen more common in the patients undergoing early RRT. The grade evidence generated was low grade for most of the outcomes.

Studies exploring the initiation strategies for RRT have shown conflicting results. Early initiation of RRT, theoretically may allow for better control of fluid and electrolyte status, fasten removal of uremic toxins and prevent complications like gastric hemorrhage and metabolic encephalopathy [20]. On the other hand a delayed strategy of RRT initiation may give sufficient time for spontaneous patient recovery and may avoid the need for RRT, thus minimizing risk associated with RRT [9].

Two recent trials [8, 9] have also shown conflicting results regarding timing of initiation of RRT. Zarbock et al. [9] (ELAIN trial) reported a significant reduction of mortality over 90 days in critically ill patients with AKI undergoing RRT while in the study by Gaudry et al. [8] (AKIKI trial) the authors found no significant reduction in mortality in patients assigned to early RRT as compared to late RRT. This difference may be due to different patient’s characteristics such as inclusion of more ill patients in the ELAIN trial as compared to that in AKIKI trial (SOFA 16 versus SOFA 11) [21]. Another difference was the use of RRT modality in the two studies. In the AKIKI trial 55% of the patients received intermittent hemodialysis as RRT modality while all the patients received CRRT in ELAIN trial. However, we have done a subgroup analysis based on the modality of RRT, severity of illness and type of patients and found no difference in mortality rates among the two groups. A recent systematic review has shown a benefit of early RRT on reduction of all cause mortality [22]. However, greater heterogeneity in the studies and a combined analysis of both RCTs and non- RCTs together may have overestimated the effect. Further, on subgroup analysis based on the type of studies (RCTs versus non RCTs), authors found no statistically significant decrease in the mortality rate in RCT group.

On subgroup analysis based on risk of bias for allocation concealment we found a significant reduction in mortality (26%) in the patients assigned to early RRT. Previous studies have also shown that treatment inadequate allocation concealment may exaggerate treatment effect by 40% and unclear allocation concealment may exaggerate treatment effect by 30%.

The strength of present systematic review is:1) we have included both randomized and quasi-randomized controlled trials to strengthen the present evidence2) we have done sensitivity analysis by excluding trials with unclear and high risk of bias for allocation concealment3) we also assigned GRADE evidence to further grade the quality of evidence and recommendations.

## Conclusion

This updated meta-analysis showed no added benefit of early initiation of RRT for patients with AKI with respect to all cause mortality, dialysis dependence, and recovery of renal functions or hospital stay. The grade evidence generated was of “low quality” and there was high heterogeneity in the included trials. We need more good quality RCTs in different patient subgroups including children to further strengthen the evidence.

## Additional file

Additional file 1: Figure S1. a: Forest plot showing period -wise mortality. b: Forest plot showing subgroup; Mixed Vs surgical patients. c: Forest plot showing dialysis dependence on day 90. d: Forest plot showing recovery of renal functions by day 90. (PDF 309 kb)

## Abbreviations

AKI: Acute Kidney Injury
CRRT: Continues renal replacement therapy
IHD: Intermittent hemodialysis
RCTs: Randomized controlled trials
RRT: Renal replacement therapy
