# Supplementary material for: Early versus late initiation of renal replacement therapy in patients with acute kidney injury-a systematic review & meta-analysis of randomized controlled trials
Source: BMC Nephrol. 2017 Feb 28;18:78. doi: 10.1186/s12882-017-0486-9 (PMC5331682; doi:10.1186/s12882-017-0486-9)
Supplement: Additional file 1: Figure S1. — a: Forest plot showing period -wise mortality. b: Forest plot showing subgroup; Mixed Vs surgical patients. c: Forest plot showing dialysis dependence on day 90. d: Forest plot showing recovery of renal functions by day 90. (PDF 309 kb) [file 12882_2017_486_MOESM1_ESM.pdf]

| Study or Subgroup | Early RRT Events | Early RRT Total | Late RRT Events | Late RRT Total | Weight | Odds Ratio M-H, Random, 95% CI |
|---|---|---|---|---|---|---|
| **1.2.1 Overall ICU mortality** | | | | | | |
| Bouman 2002 | 23 | 70 | 10 | 36 | 3.4% | 1.27 [0.53, 3.08] |
| Combes 2015 | 49 | 112 | 44 | 112 | 6.2% | 1.20 [0.71, 2.05] |
| Wald 2015 | 13 | 48 | 16 | 52 | 3.5% | 0.84 [0.35, 1.99] |
| **Subtotal (95% CI)** | | **230** | | **200** | **13.1%** | **1.12 [0.75, 1.68]** |
| Total events | 85 | | 70 | | | |
| Heterogeneity: Tau² = 0.00; Chi² = 0.59, df = 2 (P = 0.75); I² = 0% | | | | | | |
| Test for overall effect: Z = 0.57 (P = 0.57) | | | | | | |
| **1.2.2 Overall hospital mortality** | | | | | | |
| Bouman 2002 | 31 | 70 | 14 | 36 | 3.8% | 1.25 [0.55, 2.83] |
| Combes 2015 | 50 | 112 | 44 | 112 | 6.2% | 1.25 [0.73, 2.12] |
| Durmaz 2002 | 1 | 21 | 7 | 23 | 0.7% | 0.11 [0.01, 1.03] |
| Jamale 2013 | 21 | 102 | 13 | 106 | 4.2% | 1.85 [0.87, 3.94] |
| Pursnani 1997 | 4 | 18 | 5 | 17 | 1.4% | 0.69 [0.15, 3.15] |
| Wald 2015 | 16 | 48 | 19 | 48 | 3.7% | 0.76 [0.33, 1.76] |
| **Subtotal (95% CI)** | | **371** | | **342** | **20.1%** | **1.08 [0.70, 1.68]** |
| Total events | 123 | | 102 | | | |
| Heterogeneity: Tau² = 0.09; Chi² = 7.33, df = 5 (P = 0.20); I² = 32% | | | | | | |
| Test for overall effect: Z = 0.36 (P = 0.72) | | | | | | |
| **1.2.3 Overall day 14 mortality** | | | | | | |
| Sugahara 2004 | 2 | 14 | 12 | 14 | 0.8% | 0.03 [0.00, 0.23] |
| **Subtotal (95% CI)** | | **14** | | **14** | **0.8%** | **0.03 [0.00, 0.23]** |
| Total events | 2 | | 12 | | | |
| Heterogeneity: Not applicable | | | | | | |
| Test for overall effect: Z = 3.32 (P = 0.0009) | | | | | | |
| **1.2.4 Overall day 30 mortality** | | | | | | |
| Bouman 2002 | 20 | 70 | 9 | 36 | 3.3% | 1.20 [0.48, 3.00] |
| Combes 2015 | 40 | 112 | 40 | 112 | 6.0% | 1.00 [0.58, 1.73] |
| Durmaz 2002 | 1 | 21 | 7 | 23 | 0.7% | 0.11 [0.01, 1.03] |
| Gaudry 2016 | 129 | 312 | 134 | 308 | 8.8% | 0.92 [0.67, 1.26] |
| Payen 2009 | 21 | 37 | 19 | 39 | 3.3% | 1.38 [0.56, 3.41] |
| Zarbock 2016 | 44 | 112 | 65 | 119 | 6.3% | 0.54 [0.32, 0.91] |
| **Subtotal (95% CI)** | | **664** | | **637** | **28.4%** | **0.85 [0.60, 1.20]** |
| Total events | 255 | | 274 | | | |
| Heterogeneity: Tau² = 0.07; Chi² = 8.36, df = 5 (P = 0.14); I² = 40% | | | | | | |
| Test for overall effect: Z = 0.92 (P = 0.36) | | | | | | |
| **1.2.5 Overall day 60 mortality** | | | | | | |
| Combes 2015 | 48 | 112 | 42 | 112 | 6.2% | 1.25 [0.73, 2.13] |
| Gaudry 2016 | 150 | 312 | 153 | 308 | 8.8% | 0.94 [0.68, 1.29] |
| Zarbock 2016 | 43 | 112 | 60 | 119 | 6.3% | 0.61 [0.36, 1.03] |
| **Subtotal (95% CI)** | | **536** | | **539** | **21.3%** | **0.90 [0.64, 1.27]** |
| Total events | 241 | | 255 | | | |
| Heterogeneity: Tau² = 0.04; Chi² = 3.58, df = 2 (P = 0.17); I² = 44% | | | | | | |
| Test for overall effect: Z = 0.59 (P = 0.55) | | | | | | |
| **1.2.6 Overall day 90 mortality** | | | | | | |
| Combes 2015 | 51 | 112 | 43 | 112 | 6.2% | 1.34 [0.79, 2.28] |
| Wald 2015 | 18 | 48 | 19 | 52 | 3.8% | 1.04 [0.46, 2.35] |
| Zarbock 2016 | 44 | 112 | 65 | 119 | 6.3% | 0.54 [0.32, 0.91] |
| **Subtotal (95% CI)** | | **272** | | **283** | **16.3%** | **0.90 [0.49, 1.64]** |
| Total events | 113 | | 127 | | | |
| Heterogeneity: Tau² = 0.19; Chi² = 5.98, df = 2 (P = 0.05); I² = 67% | | | | | | |
| Test for overall effect: Z = 0.36 (P = 0.72) | | | | | | |
| **Total (95% CI)** | | **2087** | | **2015** | **100.0%** | **0.92 [0.75, 1.11]** |
| Total events | 819 | | 840 | | | |
| Heterogeneity: Tau² = 0.09; Chi² = 39.36, df = 21 (P = 0.009); I² = 47% | | | | | | |
| Test for overall effect: Z = 0.87 (P = 0.39) | | | | | | |
| Test for subgroup differences: Chi² = 12.19, df = 5 (P = 0.03), I² = 59.0% | | | | | | |

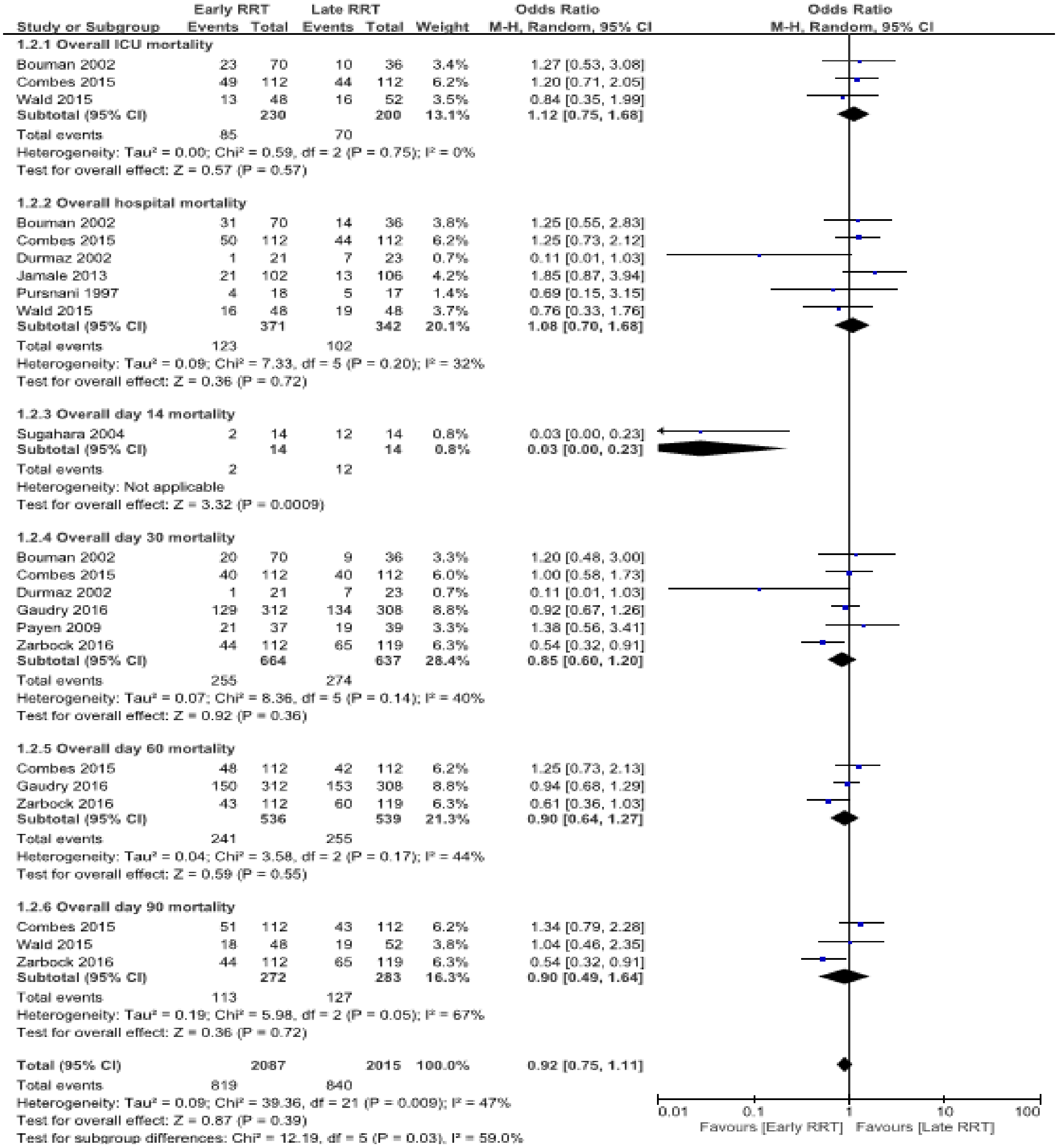


**Figure S 1 a: Period -wise mortality**

| Study or Subgroup | Early RRT Events | Early RRT Total | Late RRT Events | Late RRT Total | Weight | Risk Ratio M-H, Random, 95% CI |
|---|---|---|---|---|---|---|
| **2.1.1 Mixed surgical and medical patients** | | | | | | |
| Bouman et al. | 20 | 70 | 9 | 36 | 7.2% | 1.14 [0.58, 2.25] |
| Gaudry 2016 | 150 | 312 | 153 | 308 | 22.6% | 0.97 [0.82, 1.14] |
| Jamale 2013 | 21 | 102 | 13 | 106 | 7.8% | 1.68 [0.89, 3.17] |
| Payen 2009 | 20 | 37 | 19 | 39 | 12.4% | 1.11 [0.72, 1.72] |
| Pursnani 1997 | 4 | 18 | 5 | 17 | 3.1% | 0.76 [0.24, 2.35] |
| Wald 2015 | 18 | 48 | 19 | 52 | 10.4% | 1.03 [0.62, 1.71] |
| Zarbock 2016 | 44 | 112 | 65 | 119 | 17.8% | 0.72 [0.54, 0.95] |
| **Subtotal (95% CI)** | | **699** | | **677** | **81.3%** | **0.96 [0.81, 1.14]** |
| Total events | 277 | | 283 | | | |
| Heterogeneity: Tau² = 0.01; Chi² = 7.91, df = 6 (P = 0.25); I² = 24% | | | | | | |
| Test for overall effect: Z = 0.44 (P = 0.66) | | | | | | |
| **2.1.2 Surgical Patients** | | | | | | |
| Combes 2015 | 40 | 112 | 40 | 112 | 15.2% | 1.00 [0.70, 1.42] |
| Durmaz 2002 | 1 | 21 | 7 | 23 | 1.1% | 0.16 [0.02, 1.17] |
| Sugahara 2004 | 2 | 14 | 12 | 14 | 2.4% | 0.17 [0.05, 0.61] |
| **Subtotal (95% CI)** | | **147** | | **149** | **18.7%** | **0.35 [0.08, 1.60]** |
| Total events | 43 | | 59 | | | |
| Heterogeneity: Tau² = 1.38; Chi² = 10.24, df = 2 (P = 0.006); I² = 80% | | | | | | |
| Test for overall effect: Z = 1.35 (P = 0.18) | | | | | | |
| **Total (95% CI)** | | **846** | | **826** | **100.0%** | **0.93 [0.75, 1.15]** |
| Total events | 320 | | 342 | | | |
| Heterogeneity: Tau² = 0.05; Chi² = 17.93, df = 9 (P = 0.04); I² = 50% | | | | | | |
| Test for overall effect: Z = 0.67 (P = 0.50) | | | | | | |
| Test for subgroup differences: Chi² = 1.67, df = 1 (P = 0.20), I² = 40.2% | | | | | | |

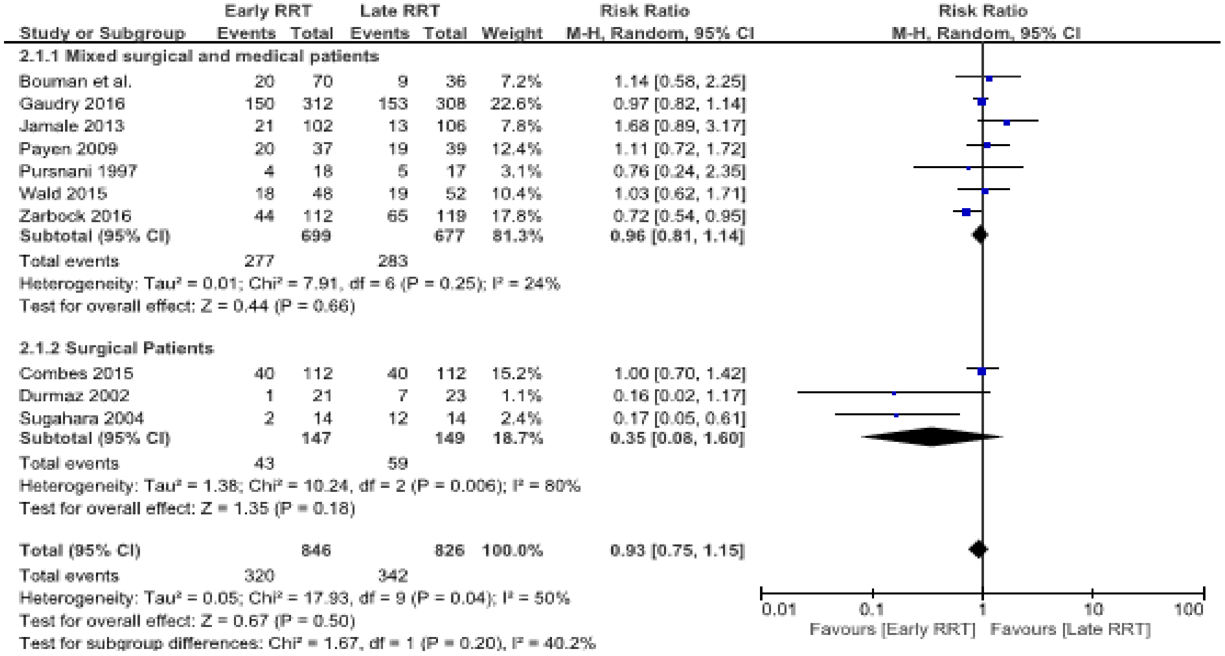


**Figure S 1 b: Subgroup; Mixed Vs surgical patients**

| Study or Subgroup | Early RRT Events | Early RRT Total | Late RRT Events | Late RRT Total | Weight | Risk Ratio M-H, Fixed, 95% CI |
|---|---|---|---|---|---|---|
| Jamale 2013 | 5 | 102 | 5 | 106 | 33.6% | 1.04 [0.31, 3.48] |
| Wald 2015 | 1 | 48 | 2 | 52 | 13.2% | 0.54 [0.05, 5.78] |
| Zarbock 2016 | 9 | 112 | 8 | 119 | 53.2% | 1.20 [0.48, 2.99] |
| **Total (95% CI)** | | **262** | | **277** | **100.0%** | **1.06 [0.53, 2.12]** |
| Total events | 15 | | 15 | | | |

Heterogeneity: $Chi^2 = 0.38$, df = 2 (P = 0.83); $I^2 = 0\%$
Test for overall effect: Z = 0.16 (P = 0.88)

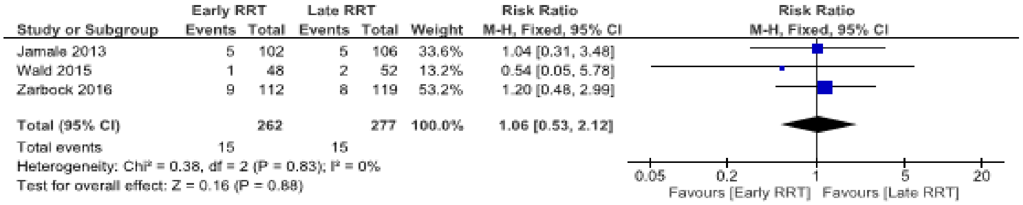


**Fig S1 c: Dialysis dependence on day 90**

| Study or Subgroup | Early RRT Events | Early RRT Total | Late RRT Events | Late RRT Total | Weight | Risk Ratio M-H, Random, 95% CI |
|---|---|---|---|---|---|---|
| Bouman 2002 | 50 | 70 | 27 | 36 | 34.1% | 0.95 [0.75, 1.21] |
| Combes 2015 | 61 | 112 | 69 | 112 | 35.4% | 0.88 [0.71, 1.11] |
| Zarbock 2016 | 60 | 112 | 46 | 119 | 30.5% | 1.39 [1.04, 1.84] |
| **Total (95% CI)** | | **294** | | **267** | **100.0%** | **1.04 [0.80, 1.35]** |
| Total events | 171 | | 142 | | | |

Heterogeneity: $Tau^2 = 0.04$; $Chi^2 = 6.57$, $df = 2$ ($P = 0.04$); $I^2 = 70\%$

Test for overall effect: $Z = 0.30$ ($P = 0.77$)

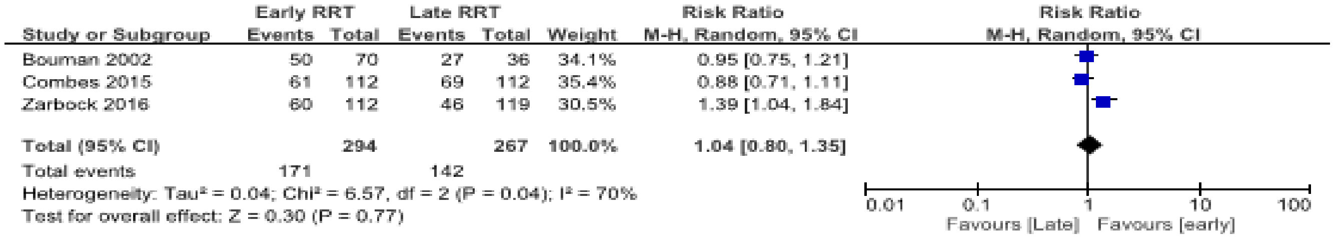


**Fig S 1 d: Recovery of renal functions by day 90**
